# Prediction of gastrointestinal disease with over-the-counter diarrheal remedy sales records in the San Francisco Bay Area

**DOI:** 10.1186/1472-6947-10-39

**Published:** 2010-07-20

**Authors:** Michelle L Kirian, June M Weintraub

**Affiliations:** 1 City and County of San Francisco. Department of Public Health, Environmental Health. 1390 Market Street suite 910, San Francisco, California 94102, USA

## Abstract

**Background:**

Water utilities continue to be interested in implementing syndromic surveillance for the enhanced detection of waterborne disease outbreaks. The authors evaluated the ability of sales of over-the-counter diarrheal remedies available from the National Retail Data Monitor to predict endemic and epidemic gastrointestinal disease in the San Francisco Bay Area.

**Methods:**

Time series models were fit to weekly diarrheal remedy sales and diarrheal illness case counts. Cross-correlations between the pre-whitened residual series were calculated. Diarrheal remedy sales model residuals were regressed on the number of weekly outbreaks and outbreak-associated cases. Diarrheal remedy sales models were used to auto-forecast one week-ahead sales. The sensitivity and specificity of signals, generated by observed diarrheal remedy sales exceeding the upper 95% forecast confidence interval, in predicting weekly outbreaks were calculated.

**Results:**

No significant correlations were identified between weekly diarrheal remedy sales and diarrhea illness case counts, outbreak counts, or the number of outbreak-associated cases. Signals generated by forecasting with the diarrheal remedy sales model did not coincide with outbreak weeks more reliably than signals chosen randomly.

**Conclusions:**

This work does not support the implementation of syndromic surveillance for gastrointestinal disease with data available though the National Retail Data Monitor.

## Background

Syndromic surveillance has received much attention as a method for health departments to accelerate the detection of, the reaction to, or the confirmation of disease outbreaks [1,2]. After the publication of reports suggesting that monitoring over-the-counter drug sales might have given advance notice of the 1993 outbreak of cryptosporidiosis in Milwaukee [3-5], federal agencies began to make explicit recommendations that water utilities and health departments consider implementing over-the-counter syndromic surveillance for enhanced waterborne outbreak detection [6-8]. However, the ability of over-the-counter syndromic surveillance to enhance the detection of waterborne disease outbreaks has not been adequately demonstrated [9].

In the San Francisco Bay Area, drinking water is provided by the San Francisco Public Utilities Commission (SFPUC) to 2.4 million customers in four counties. Supported by the SFPUC, the San Francisco Department of Public Health's Water Epidemiology Program maintains regional, distribution system-wide cryptosporidiosis surveillance. To clarify the validity and representativeness of sales of over-the-counter diarrheal remedies available through the National Retail Data Monitor (NRDM) for prospective outbreak detection, we sought to determine if these data are related to known outbreaks of infectious gastrointestinal illness in the drinking water service area [10].

## Methods

County and state agencies receive reports of individual gastrointestinal cases as well as infectious disease outbreaks. Title 17 of the California Code of Regulations mandates case reporting of specified diagnosed diseases as well as outbreaks of any disease to local health departments by health care providers [11]. Health departments may also become aware of outbreaks through follow-up with individual reported cases, citizen complaints and other modes. The definition of an outbreak differs by disease but typically entails a group of related cases for which a common source is identified or suspected; outbreaks may include as few as two cases.

Reports of cases of gastrointestinal disease from 2001-2007 among residents were requested from each of the county health departments in the drinking water service area. Data were transmitted in electronic formats from three adjacent counties. Reports for each case included etiology, date of report to the health department, gender, age, city and county.

Electronic records of outbreak data for all three participating counties were provided by the California Department of Public Health which receives outbreak reports following county and state health department outbreak investigations. These data were combined and reconciled with electronic records and records which were manually extracted from paper files from two of the participating county health departments. For each outbreak, information on etiology, number of cases, date of symptoms onset for the first and last cases, affected counties, and whether the outbreak occurred in an institutional setting such as a nursing home was provided. Outbreaks of reportable diseases as well as outbreaks of diseases that are not reportable as listed in Title 17 were included. Individual cases reportable under Title 17 associated with any outbreak may be included in the diarrhea case dataset; however, sufficient information was not available to link the outbreak and case datasets. The Committee on Human Research at the University of California, San Francisco approved the study protocol.

Over-the-counter drug sales records were purchased from the NRDM [10]. Records for the years 2005-2007 were provided as an electronic file. Records for years 2003-2004 were downloaded using the NRDM web interface. NRDM over-the-counter drug sales records are divided into 18 categories based on common use, form and whether intended for adult or pediatric populations. NRDM drug categories are: diarrhea remedies, anti-fever adult, anti-fever pediatric, bronchial remedies, baby/child electrolytes, chest rubs, cold relief adult liquid, cold relief adult tablet, cold relief pediatric liquid, cold relief pediatric tablet, cough syrup adult liquid, cough adult tablet, cough syrup pediatric liquid, cough/cold, hydrocortisones, nasal product internal, throat lozenges, and thermometers. Sales are based on the number of units sold regardless of the package size. Daily total sales are available for both all units sold by category and units sold by category excluding units for which discounts or other promotions were offered during the reporting period. NRDM provides information on the number of stores enrolled and reporting; from 2005 through 2007 approximately 47% of the stores enrolled to report anti-diarrhea drug sales actually reported (number of stores enrolled per week: 1389 -1706; number of stores reporting: 592-836).

Our analysis variable was the proportion of non-promotional diarrhea remedy sales to sales of non-promotional drugs for all categories combined (Diarrheal Remedy Sales). Diarrheal remedies are products taken for the relief of diarrhea and include bismuth, attapulgite, subsalicylate, and loperaminde hydrochloride products. Sales records of diarrheal remedies were available for the entire study area from July 2003 through 2007. Proportion sales were used instead of counts to control for unknown confounders such as changes in store hours.

Diarrheal Remedy Sales, and gastrointestinal case and outbreak data were aggregated by week for analysis. Diarrheal Remedy Sales were aggregated by week of sale, cases by week of report to the health department and outbreaks by week of onset of first outbreak-associated case. Data were divided into three parts for model building, model validation, and forecasting.

We used methods developed by Box and Jenkins to build autoregressive integrated moving average (ARIMA) models [12]. Estimates of model parameters were obtained through the method of least squares. All analyses were performed using SAS version 9.1 (SAS Institute Inc., Cary, NC, USA). Using Proc ARIMA, following either pre-whitening or double pre-whitening, Diarrheal Remedy Sales were cross correlated with the number of diarrhea cases in the same week and with weekly counts lagged one to 19 weeks before and after.

The relationship between Diarrheal Remedy Sales and gastrointestinal outbreaks was examined graphically and through regression. Because a 2006 report by Edge and colleagues [13] suggested that over-the-counter drug sales are sensitive to viral infection, specifically Norovirus, Diarrheal Remedy Sales were compared to outbreaks of all etiologies combined and to outbreaks of Norovirus alone. Furthermore, as institutionalized populations, such as those in a nursing home, may not purchase drugs from over-the-counter drug vendors in the same way as the non-institutionalized population, analyses were repeated excluding outbreaks that occurred in an institutional setting. Diarrheal Remedy Sales univariate model residuals were regressed on the number of outbreaks and on outbreak-associated cases per week.

The univariate Diarrheal Remedy Sales ARIMA model was used to auto-forecast sales for 105 weeks with weekly model updating (one week ahead forecasting). Signals were generated when actual observations exceeded the upper 95% confidence limit. An outbreak week was any week when one or more outbreaks started that week or prior to that week but ended that week or later. Model sensitivity was calculated as the number of outbreak weeks with a signal divided by the total number of outbreak weeks. Specificity was calculated as the total number of weeks without a signal and no detected outbreaks divided by the total number of weeks without an outbreak. Calculations were done with all outbreaks and repeated in subsets of only larger outbreaks with 50 or more or 100 or more cases. To evaluate if model derived alerts identified outbreak weeks more reliably than randomly chosen alerts, sensitivity and specificity calculations were repeated for three sets of randomly chosen dates.

## Results

**Figure 1:**
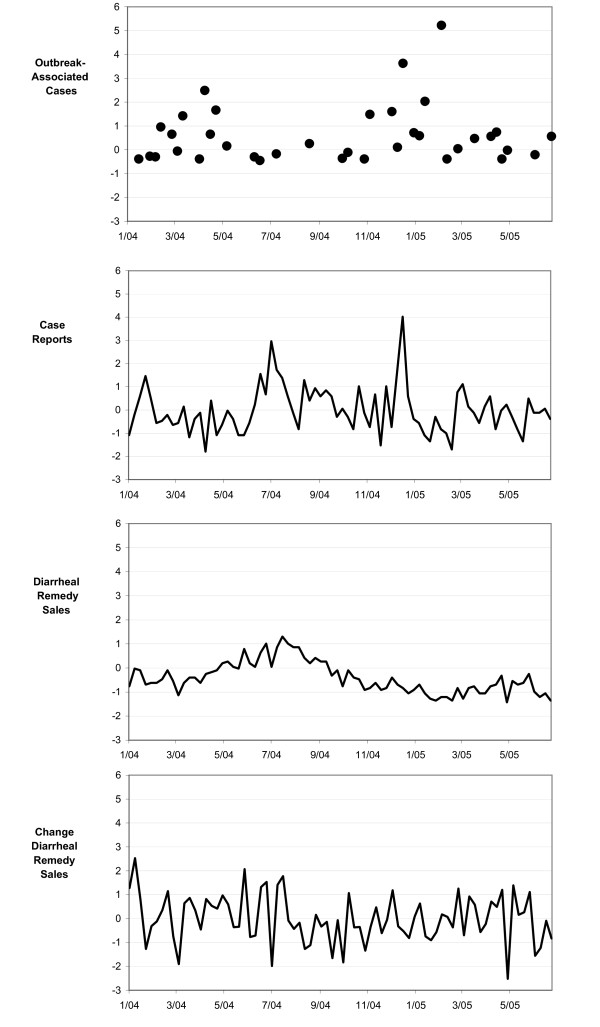
**Plots of Outbreak-Associated Gastrointestinal Cases, Individual Gastrointestinal Cases, Diarrheal Remedy Sales, and Differenced Diarrheal Remedy Sales**. Standardized weekly counts of gastrointestinal outbreak-associated cases, diarrheal illness case reports, Diarrheal Remedy Sales and differenced Diarrheal Remedy Sales in three San Francisco Bay Area Counties from January 2004 to July 2005. All data aggregated to the first Sunday of week. Diarrheal Remedy Sales are aggregated by week of sale, cases by week of report to the health department and outbreak cases by week of onset of the first associated case. Vertical axes are measured in standard deviations.

Diarrheal case data were fit with a first order autoregressive model and Diarrheal Remedy Sales with an integrated first order moving average model (Case ARIMA(1,0,0): parameter estimate 0.33, T-ratio 3.09; Diarrheal Remedy Sales ARIMA(0,1,1): 0.4, 4.42). Figure 1 presents time series plots of the outbreak -associated gastrointestinal cases, individual gastrointestinal cases, Diarrheal Remedy Sales and differenced Diarrheal Remedy Sales.

From July 2003 through December 2007, there were 233 gastrointestinal outbreaks (Table 1). Most reported outbreaks were caused by Norovirus or by an unknown etiology of which many were suspected of being Norovirus. More Norovirus outbreaks were reported in each of 2006 and 2007 than previous years. Norovirus outbreaks were also larger than outbreaks of other diseases with a mean number of outbreak-associated cases of 30. The largest outbreak was of Norovirus at 153 cases. Thirty percent of outbreaks occurred in an institutional setting.

**Table 1:** Gastrointestinal Outbreak and Case Characteristics in the San Francisco Bay Area, July 2003 through December 2007

	N	Outbreak-Associated Cases*				N
		Maximum	Median	Mean		
All Disease Outbreaks	233	153	19	25	Case Etiology	
Outbreaks of Reportable Diseases	31	65	12	16	campylobacteriosis	3316
Outbreaks of Not-Reportable Diseases	202	153	21	27	cryptosporidiosis	2210
Study Period					salmonellosis	2187
Model (6/29/03 to 7/2/05)†	71	110	15	23	giardiasis	1739
Validation (7/3/05 to 12/31/05)	11	26	14	13	shigellosis	1002
Forecasting (1/1/06 to 12/30/07)	154	153	21	27	amoebiasis	512
Outbreak Etiology					hepatitis A	152
norovirus	144	153	24	30	Escherichia *coli *infection	151
unknown	41	80	18	21	vibriosis	118
salmonellosis	17	65	13	22	typhoid	62
Bacillus *cereus */Clostridium *perfringens *infection	8	38	8	12	listeriosis	39
Escherichia *coli *infection	4	18	11	11	yersiniosis	26
scombroid poisoning	3	7	5	5	legionellosis	17
bacterial toxin poisoning	3	22	4	10	ciguatera poisoning	5
chemical toxin poisoning	2	4	3	3		
vibriosis	2	27	21	21		
ciguatera poisoning	2	3	3	3		
hepatitis A	1	2	2	2		
trichinosis	1	2	2	2		
cryptosporidiosis	1	16	16	16		
yersiniosis	1	1	1	1		
giardiasis	1	14	14	14		
rotavirus	1	6	6	6		
campylobacter	1	3	3	3		

*Number of cases not available for five outbreaks (3 salmonellosis, 2 norovirus) between January 1, 2006 and January 1, 2008.

† Modeling period was shorter --Jan 4, 2004 to Jul 2, 2005-- for Univariate Case Modeling and Cross Correlation Analysis.

σ Outbreak and case data sets are not mutually exclusive or encompassing.

In the forecasting period, January 1, 2006 to January 1, 2008, there were 154 outbreaks; 20 with 50 or more, three of these with 100 or more cases. Table 2 lists details for outbreaks with 50 or more cases. Table 1 provides the number and size of outbreaks by study period.

**Table 2:** Gastrointestinal Outbreaks with 50 or More Cases in the San Francisco Bay Area, January 2006 through December 2007

Etiology	Cases	First Onset	Last Onset	Institutional	Over-the-Counter Drug ARIMA (0,1,1) Signal
Norovirus	101	1/25/06		Yes	Yes
Unknown/Norovirus	60	4/18/06		No	Yes
Unknown/Norovirus	62	4/24/06		Yes	Yes
Norovirus	107	4/25/06	5/2/2006	No	No
Unknown/Norovirus	55	4/26/06		No/Unknown	Yes
Norovirus	50	5/8/06		Yes	Yes
Norovirus	81	10/26/06		Yes	No
Norovirus	86	11/23/06		Yes	No
Norovirus	72	11/30/06		Yes	No
Norovirus	63	11/30/06		Yes	No
Unknown	80	12/7/06		Yes	No
Unknown	61	12/7/06		No/Unknown	No
Norovirus	76	1/3/07		Yes	No
Norovirus	60	1/8/07		Yes	No
Norovirus	92	7/13/07	7/17/07	No	No
Norovirus	153	8/3/07	8/17/07	No/Unknown	No
Norovirus	51	9/15/07	9/19/07	No/Unknown	No
Norovirus	52	12/20/07	1/1/08	Yes	No
Norovirus	52	12/22/07		Yes	No
Norovirus	76	12/22/07	1/15/08	No/Unknown	No

Abbreviations: ARIMA(0,1,1) First Order Integrated Moving Average

From 2004 through 2007 there were 11,536 reported gastrointestinal cases. The majority of cases were of campylobacteriosis, cryptosporidiosis, salmonellosis, giardiasis, shigellosis and amoebiasis (Table 1). More cases were reported among children under 5 than for any other age group; incidence of gastrointestinal illness was similar across other ages. Sixty-one percent of cases were male. Although we collected case data from 2001 through 2007, there were abrupt changes in reporting at the start of 2004; review of the average number of cases reported by day of the week and year showed consistent lower overall reporting by day for 2004 through 2007 as compared to earlier data potentially indicating a change in surveillance protocols. As these county level changes persisted in aggregated regional data, the data were restricted to 2004 and later for analysis.

From July 2003 through December 2007, the proportion of diarrheal remedy sales to total drug sales ranged from 0.016 to 0.083 with an average of 0.044 and standard deviation of 0.014. Sales of diarrhea remedies ranged from 1216 to 3512 unit sales per week with an average of 2435 and standard deviation 441.

**Figure 2:**
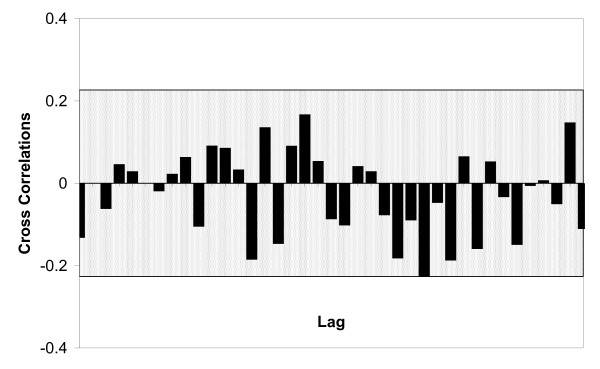
**Cross Correlations Between Diarrheal Remedy Sales and Diarrheal Illnesses**. Diarrheal Remedy Sales and diarrheal illness case reports cross correlations at time lags from zero to 19 weeks. No significant correlations, bars exceeding the 95% confidence interval (shaded), were found.

No significant correlation at any lag was found between Diarrheal Remedy Sales and diarrheal cases (Figure 2). Furthermore, regression analysis of the Diarrheal Remedy Sales univariate model residuals did not reveal an association between the weekly number of outbreaks or outbreak-associated cases and Diarrheal Remedy Sales when all outbreaks data were included or when restricted to Norovirus and/or non-institutional outbreaks.

Four signals were generated by the Diarrheal Remedy Sales model (on the weeks of 6/11/06, 1/29/06, 10/15/06 and 6/10/07). Four of the 20 outbreaks with 50 or more and one of three with 100 or more cases started during or lasted through a week with a signal. The two outbreaks with 100 or more cases without signals were both non-institutional Norovirus outbreaks with steep epidemic curves. Sensitivity for all outbreaks and for outbreaks with 50 or more or 100 or more cases was low and specificity high (Table 3). The sensitivity and specificity of the model was identical to a random selection of three sets of four signals, further supporting the conclusion that any relationship between Diarrheal Remedy Sales and gastrointestinal illness is spurious.

**Table 3:** Sensitivity and Specificity of Diarrheal Remedy Sales Model-Generated and Randomly Selected Signals

	All Outbreaks		Outbreaks with ≥ 50 Cases		Outbreaks with ≥ 100 Cases	
	Sensitivity	Specificity	Sensitivity	Specificity	Sensitivity	Specificity
Over-the-counter drug IMA(1) Signal (6/11/06, 1/29/06, 10/15/06, 6/10/07)*	4% (4/94)	100% (11/11)	4% (1/26)	97% (76/79)	14% (1/7)	97% (95/98)
Random Signals 1 (1/29/06, 6/11/06, 9/9/07, 2/18/07)*	4% (4/94)	100% (11/11)	8% (2/26)	97% (77/79)	14% (1/7)	97% (95/98)
Random Signals 2 (2/12/06, 7/9/06, 12/3/06, 9/23/07)*	3% (3/94)	91% (10/11)	4% (1/26)	97% (76/79)	0% (0/7)	96% (94/98)
Random Signals 3 (5/21/06, 1/3/07, 9/16/07, 12/9/07)*	3% (3/94)	100%(11/11)	4% (1/26)	97% (77/79)	0% (0/7)	97% (95/98)

* First day of week for each model generated and randomly generated signal.

## Discussion

NRDM Diarrheal Remedy Sales did not predict outbreaks of gastrointestinal disease or correlate with individual cases of diarrheal illness. Signals generated by the Diarrheal Remedy Sales model did not coincide with outbreak weeks more reliably than signals chosen randomly.

To generate Diarrheal Remedy Sales signals we employed ARIMA modeling and forecasting. Time series modeling, including ARIMA, has a long history of use in econometrics and statistical quality control [14,15]. More recently it has been adopted by public health practitioners to model subjects such as influenza and hospital admissions, weather and suicides, and gun bans and homicides [16-18]. Time series modeling accounts for autocorrelation, trend and seasonality which when present in data can cause ordinary regression techniques to present spurious variance estimates and incorrect inference.

***Over-The-Counter Anti-Diarrheal Drug Sales and Surveillance:***

In the aftermath of the 1993 Milwaukee waterborne cryptosporidiosis outbreak in which thousands were sickened it was reported that sales of over-the-counter anti-diarrheal and anti-cramping drugs at one pharmacy increased by a factor of 17 to 20 as compared to the same period in the previous year [3]. This finding, supported by similar anecdotal reports stimulated the push for the implementation of waterborne disease surveillance with over-the-counter drug sales [19]. However, a later review of the feasibility and timeliness of surveillance data available during that outbreak--water treatment plant effluent turbidity logs, clinical laboratory diagnosis, nursing home diarrhea rates, hospital emergency room logs, random digit dialing telephone surveys, water utility complaint logs, school absentee logs and sales of anti-diarrhea drugs--revealed a poor response rate by pharmacies and a lack of timeliness [5].

A subsequent retrospective analysis of anti-nauseants and anti-diarrhea drug sales during waterborne outbreaks of cryptosporidiosis (Battlefords, Saskatchewan), and E. coli 0157:H7 infection and campylobacteriosis (Walkerton, Ontario), found that increased over-the-counter drug sales coincided with or lagged shortly behind illness onset [4]. The authors concluded that over-the-counter drug sales trends would provide a more timely and sensitive tool than monitoring hospital emergency department visits or traditional passive laboratory based surveillance. Nonetheless, over-the-counter drug sales data limitations were noted: data from only one of three pharmacies in Battlefords and one of six in Walkerton were available and formatted appropriately for analysis.

Studies of the seasonality of over-the-counter drug sales and diarrhea illness have also contributed evidence supporting over-the-counter drug sales for enhanced gastrointestinal surveillance. In an unidentified Canadian providence, sales of anti-nauseant and anti-diarrhea over-the-counter drugs from one major retailer with 19 locations, accounting for only 12% of all pharmacies in the region, had similar seasonal temporality with reported Norovirus infections [13]. However, over-the-counter drug sales did not coincide with diarrhea due to other etiologies specifically bacterial or parasitic which are more prevalent during summer months. Similarly, electrolyte sales followed the same seasonal pattern as hospitalizations for selected pediatric diarrheal illness (Rotavirus and intestinal infections due to organisms not elsewhere classified (ICD9 008.61)) when combined with pediatric respiratory illnesses (Pneumonia, bronchopneumonia, influenza, bronchiolitis, respiratory syncytial virus) [20]. This study included very few diarrhea illness etiologies and the number of cases of each illness are not presented; the incidence of respiratory illnesses, especially seasonal influenza, is likely to greatly exceed that of diarrheal illnesses therefore obscuring the relationship between over-the-counter drug sales and diarrhea illness. The authors acknowledged that it is not possible to rule out a coincidental relationship which is driven by other phenomena.

Local and state health departments have implemented syndromic surveillance systems with over-the-counter anti-diarrhea drug sales monitoring components but few retrospective studies and no successful reports from ongoing surveillance projects are published [3,21,22]. Only one report, now antiquated, presents the progress of a functioning over-the-counter anti-diarrhea drug sales monitoring program. Similar to our results, Das et al (2005) reported that they had found no consistent relationship between over-the-counter anti-diarrhea drug sales and emergency department visits for gastrointestinal illness in New York City [22]. And, despite its availability nationwide for more than six years, no publications evaluate surveillance with NRDM over-the-counter diarrheal remedy drug sales in practice. One retrospective study presented graphs demonstrating the similar temporality of analgesic, anti-fever, anti-diarrhea and cough, and cold drugs combined and calls to the poison control center in 2003 [23]. Although our literature review did identify a number of reports suggesting that syndromic surveillance with over-the-counter anti-diarrheal drug sales could enhance traditional disease control activities, the widespread adoption of syndromic surveillance systems and the paucity of published reports on over-the-counter drug sales monitoring systems, and NRDM specifically, suggest publication bias may be present.

## Limitations

Our study had several limitations. First, there were no large regional outbreaks in our dataset and the high data variability of diarrhea remedy sales may make it difficult to discern changes resultant from relatively small increases in illness. Although we do not believe that individual early health seeking behavior such as over-the-counter drug purchases would be different when an individual's illness is part of an undetected larger outbreak, in a large outbreak the number of people pursuing over-the-counter remedies might produce a signal that is significantly above the noise in the baseline.

Over-the-counter drug sales records as provided by the NRDM have several limitations. The usability of these data could be improved if participation by enrolled stores was increased or if meta-information on participating stores such as market coverage and on the drugs included in each category were made available. While we did not find any association between gastrointestinal disease and purchases of diarrheal remedies in general, it is possible that one product or a subset of products included in this category might have coincided with known disease. Furthermore, our study was not able to assess whether improvements in over-the-counter drug sales reporting systems might enhance the performance of this type of syndromic surveillance. The use of over-the-counter drugs sales for surveillance may be prohibitive due to the cost and logistics of data collection, or the proprietary and secret nature of the data [3].

County-by-county differences in disease reporting, and aggregations of diseases with varying severities may have masked finding a true association. These aggregations could also have covered up localized Diarrheal Remedy Sales fluctuations resultant from isolated outbreaks. We therefore cannot rule out that county specific syndromic surveillance may be more sensitive than the region-wide surveillance examined in this analysis.

While studies show that over-the-counter drugs are the first option for many, health seeking behavior varies by factors including age, gender and culture [24-32]. One study examined healthcare-seeking behavior in response to diarrheal illness specifically. This survey of 351 adults reporting acute gastroenteritis (diarrhea, vomiting or both) found significant differences between those who use over-the-counter drugs and those who do not [24]. Although care should be exercised in applying these findings from Canada to the US as each have distinct health care systems, the lack of correlation that we found in our study between Diarrheal Remedy Sales and diarrheal cases could indicate that these data sources measure the occurrence of diarrhea in different populations. Similarly, high population mobility may increase the chances that Diarrheal Remedy Sales and cases are not both included in the region of study and that dispersed outbreaks may not be detected [33].

## Conclusions

This study did not support the implementation of syndromic surveillance with National Retail Data Monitor Diarrheal Remedy Sales for enhanced gastrointestinal outbreak detection of waterborne or other origins. However, we cannot exclude the possibility that NRDM data maybe useful for detecting larger outbreaks.

A secondary finding of the study was of the increasing role of Norovirus in disease outbreaks in our region. From 2004 through 2006 approximately 56% of all outbreaks were due to Norovirus infection, 15% of these occurred in institutional settings. In 2007 the proportion attributable to Norovirus rose to 73%; 65% of outbreaks in 2007 were institutional. The increased incidence of outbreaks due to Norovirus may be attributable to enhanced detection or reporting; however, similar increases were noted in North Carolina, New York and Wisconsin [34]. Especially given the proven effectiveness of existing programs [35], public health departments must carefully evaluate the efficacy and added worth of surveillance systems to avoid the possibility that increased funding for programs such as syndromic surveillance are not accompanied by cutbacks in funding for programs such as institutional Norovirus prevention, resulting in a net increase in overall morbidity [36].

## Abbreviations

The following abbreviations are used: Autoregressive Integrated Moving Average (ARIMA), National Retail Data Monitor (NRDM), and San Francisco Public Utilities Commission (SFPUC).

## Competing interests

The authors declare that they have no competing interests.

## Authors' contributions

JMW guided the study design, directed the implementation of the study, drafted portions of the manuscript, and critically reviewed the text. MLK designed and implemented the study, conducted the literature review, and was principal author of the text. JMW and MLK have read and approved the final manuscript.

## Pre-publication history

The pre-publication history for this paper can be accessed here:

http://www.biomedcentral.com/1472-6947/10/39/prepub
